# Is aripiprazole similar to quetiapine for treatment of bipolar depression? Results from meta-analysis of Chinese data

**DOI:** 10.3389/fpsyt.2022.850021

**Published:** 2022-09-09

**Authors:** Hejian Tao, Jiawei Wang, Dong Shen, Fengli Sun, Weidong Jin

**Affiliations:** ^1^Department of Psychiatry, Second Clinical College, Zhejiang Chinese Medical University, Hangzhou, China; ^2^Department of Psychiatry, 988 Hospital PLA, Jiaozuo, China; ^3^JIaxing Kangci Hospital, Jiaxing, China; ^4^Department of Psychiatry, Zhejiang Province Mental Health Center, Zhejiang Province Tongde Hospital, Hangzhou, China; ^5^Zhejiang Province Mental Health Center, Zhejiang Province Mental Health Institute, Zhejiang Province Tongde Hospital, Hangzhou, China

**Keywords:** aripiprazole, quetiapine, bipolar depression, meta-analysis, Chinese data

## Abstract

**Objective:**

To study the similarities and differences in the clinical efficacy of aripiprazole and quetiapine in Chinese patients with bipolar depression through meta-analysis. Additionally, to provide evidence of aripiprazole in treatment for bipolar depression.

**Methods:**

We searched Chinese literature related to the study of aripiprazole and quetiapine in treatment for bipolar depression, among which results such as comments, letters, reviews, and case reports were excluded. The clinical efficacy between aripiprazole and quetiapine was synthesized and discussed.

**Result:**

A total of 1,546 subjects were included in 17 studies. The random effect model was used to review the data by RevMan 5.2. The results showed that there was no significant difference in the remission rate between patients treated with aripiprazole and quetiapine evidenced by the scale used to evaluate the patients being treated for bipolar depression (221/501 vs. 193/501, Z = 1.12, *P* = 0.26). But the results also showed that the remission rate of aripiprazole with lithium carbonate was significantly higher than quetiapine with lithium carbonate in the treatment of bipolar depression (111/232 vs. 69/232, Z = 3.92, *P* < 0.0001). The results showed that the effective rate of aripiprazole was similar to quetiapine (426/572 vs. 386/572, Z = 2.70, *P* = 0.007). Overall, there was no difference in the Hamilton Rating Scale for Depression (HAMD) score between patients treated with aripiprazole and quetiapine (Z = 1.68, *P* = 0.09). The results also show that the drop-out rate of aripiprazole was similar to quetiapine in the treatment of patients with bipolar depression (Z = 1.80, *P* = 0.07).

**Conclusion:**

As an atypical antipsychotic, aripiprazole may be similar to quetiapine for treating bipolar depression with similar drop-out and higher remission rates when combined with lithium carbonate. However, the results of this study need to be read with caution given the poor quality of collected/analyzed literature.

## Introduction

Bipolar disorder (BD) is a common chronic mental disorder with depression and mania. It is a major factor in the global disease burden, with a prevalence of about 1% (1–3). Bipolar disorder has two subtypes: BD I and BD II. When the manic symptoms are manic episodes, it is classified as bipolar I, and when the symptoms are hypomania, it is bipolar II (4, 5). Patients with BD develop mania, irritability, or depression repeatedly and irregularly throughout their life, which may lead to social and occupational disability (4). Drug therapy is the main treatment for BD (6, 7), and there are already some guidelines for the treatment of bipolar disorder (8, 9).

Bipolar depression is the most common and difficult to treat phase of bipolar disorder. Antidepressants meant for unipolar depression are among the most widely used drugs, but recent data and meta-analyses indicate a lack of efficacy. Bipolar depression is an important contributor to the long-term dysfunction of persons with bipolar disorder due to psychosocial impairment, loss of work productivity, and a high rate of substance abuse. Missed and delayed diagnosis is quite prevalent due to overlapping symptoms with unipolar depression and other diagnoses (10).

The treatment for bipolar depression is not only difficult but also controversial. Therefore, there are many related clinical studies, reviews, and evidence-based medical evaluations in the treatment of bipolar depression (11–16). In the guidelines for the treatment of bipolar depression, in addition to mood stabilizers, select atypical antipsychotics are the main drugs recommended, which can even be used alone in the treatment of bipolar depression. These drugs include Lurasidone, Cariprazine, Olanzapine, and Quetiapine. These drugs have a good therapeutic effect on the acute or maintenance stage of bipolar depression (8, 13, 15, 17).

In addition to the above-mentioned drugs, the atypical antipsychotic aripiprazole, which acts almost only on dopamine receptors, is another drug worthy of attention. As a derivative of the dopamine autoreceptor agonist, Aripiprazole could be beneficial in the treatment of treatment-resistant depression (TRD) as an add-on therapy (18) and be equally useful in therapy for bipolar depression (11, 16). In a clinical study, aripiprazole was associated with beneficial effects on mood in some patients with bipolar depression (11, 16, 19). Quetiapine is considered to be one of the atypical antipsychotics for the treatment of bipolar depression and has been written into the treatment guidelines for bipolar depression (8, 12, 14, 15). An evidence-based medicine study in China suggests that the effective rate, remission rate, and symptom improvement of aripiprazole and quetiapine in the treatment of bipolar depression are almost similar, but supported by very little research literature (20). In recent years, Chinese psychiatrists have conducted more clinical studies on aripiprazole and quetiapine in the treatment of bipolar depression, further suggesting the role of aripiprazole in the treatment of bipolar depression. Therefore, this meta-analysis aims to evaluate the effects of aripiprazole and quetiapine in the treatment of bipolar depression and provide data from China on whether aripiprazole can be included in the treatment guidelines for the treatment of bipolar depression.

## Methods

### Search strategy

**Figure 1:**
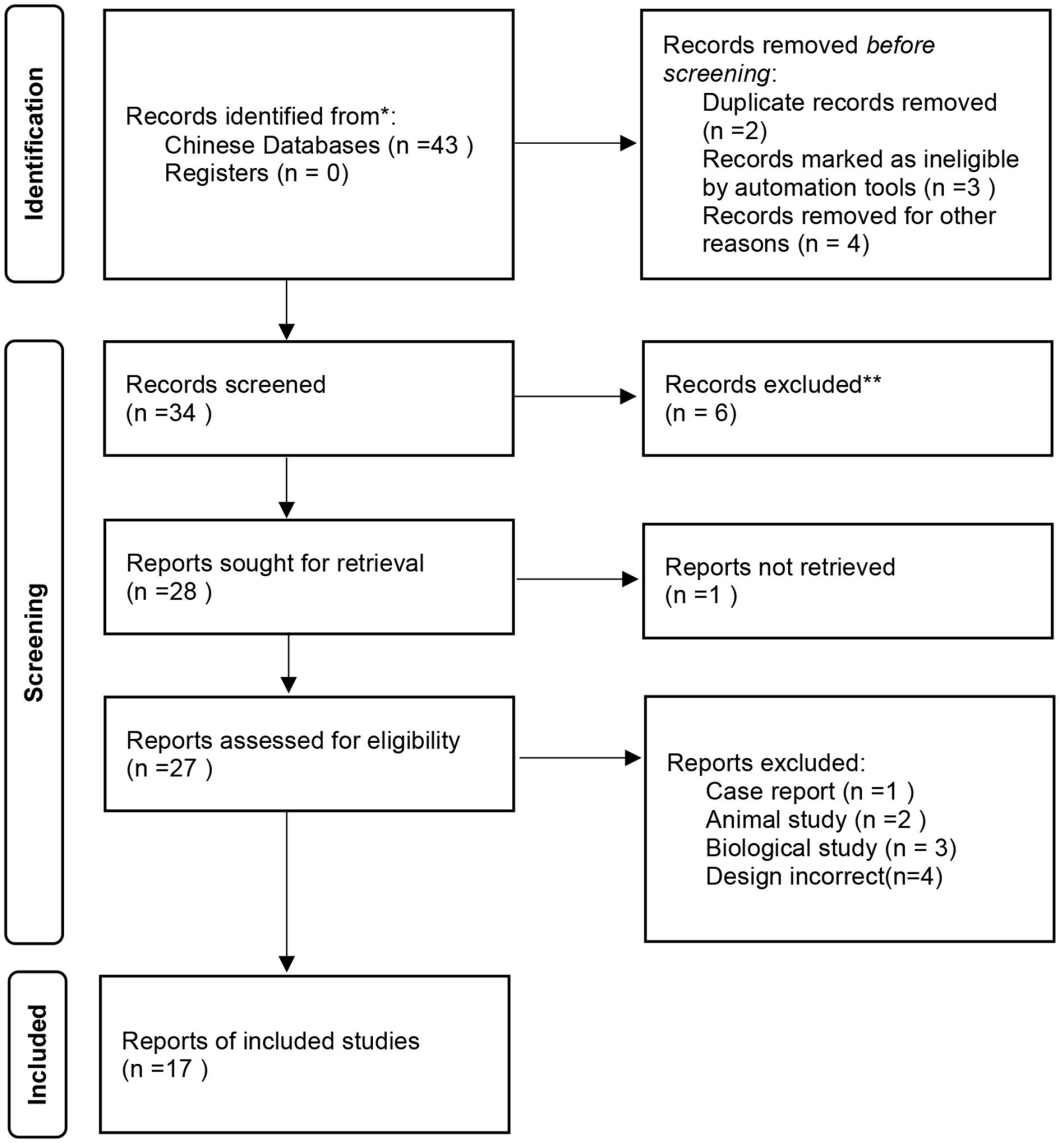
Identification, assessment and retrieval of studies.

Studies were identified by searching Chinese databases with search terms “bipolar depression”, “aripiprazole”, and, “quetiapine” in clinical trials. Only Chinese databases were scanned. They included the Chinese Biomedical Database (CBM), China National Knowledge Infrastructure (CNKI), WanFang, and the Chinese Social Sciences Citation Index (VIP). The search included the period 2000–2021. However, articles that had incomplete or unidentified data were excluded, as well as abstracts, reviews, case reports, letters, and duplicate publications according to the 2020 PRISMA guide (21). Finally, 17 studies were included in the meta-analysis (see Figure 1).

### Quality assessment

Two psychiatrists reviewed each included article independently. The Cochrane Collaboration's tool was used for assessing the risk of bias (22). Differences in article quality were discussed to reach an agreeable final score. The following information was extracted: first author, publication year, the sample size, study population, assessment tools, the number of people who had anxiety and depression, and comparison (yes or no).

### Data analysis

The primary outcome in all the trials was a change from baseline in the depression scale at the last assessment and this was also our criterion. The difference in change in scores between each drug group was noted. Where standard deviations (SD) for score changes were not available, the median SD from those trials where SD was reported was used. We also examined outcomes by response rates, defined as the proportion of subjects achieving >50% improvement, and remission rates defined as the proportion of subjects achieving HAMD of 7 at an endpoint. These endpoint definitions were homogeneous in all trials. The between-treatment comparisons were estimated using the relative ratio (RR) and 95% confidence intervals (95% CI) for the binary variables (remission and response) and the mean difference and 95% CI for the HAMD. Since it was considered likely a priori that not all trials would produce exactly equal underlying effect sizes, a random-effects model was considered preferable to a fixed-effects model. The random-effects model incorporates both within-study and between-study variance into the estimate of average treatment effects and is therefore usually more realistic that the fixed-effects model. We also performed a sensitivity analysis to assess the source of heterogeneity by excluding the aripiprazole studies, as they were negative on the primary outcome.

### Statistical analysis

All statistical analyses were performed using RevMan 5.2. The *P*-value for the overall effect <0.05 was statistically significant and I^2^ was heterogeneity of all involved studies, which was lower than 50% with an acceptable heterogeneity. The fixed-effects and random-effects models were used according to I^2^.

### Assessment of publication bias

Assessment of publication bias was investigated for each of the pooled study groups mainly using Egger's linear regression test. As a supplement approach, Begg's rank correlation was also applied to assess the potential publication bias. When *P* < 0.05, it was considered that there was no publication bias in the study.

## Results

### Study characteristic

A total of 17 comparison studies, with 773 cases in the aripiprazole group and 773 cases in the quetiapine group, met the inclusion criteria and were included in the final meta-analysis. Four studies reported the HAMD scale without effective and remission rates. Ten studies compared remission rates and twelve studies compared effective rates. Five studies compared aripiprazole and quetiapine, four studies compared aripiprazole and quetiapine based on valproate sodium. Further, five studies compared aripiprazole and quetiapine based on lithium carbonate. The sample size of the studies ranged from 15 to 81 cases in each group. Assessment tools for therapeutic effectiveness used in the studies were HAMD and CGI. The main features of the 17 articles are summarized in Table 1 (23–39).

**Table 1:** Characteristics of studies included in the meta-analysis.

**Author** **(year)**	**Design**	**Aripiprazole group (AG) cases**	**Remission/Effective cases in AG**	**Quetiapine group (QG) cases**	**Remission/Effective cases in QG**	**Drugs**
Li et al. (23)	Comparison	40	11/23	40	11/23	A/Q
Yu et al. (24)	Comparison	75	29/40	75	39/37	A/Q
Zhang et al. (25)	Comparison	56	16/30	56	40/13	A/Q
Li and Jiang (26)	Comparison	48	25/40	48	23/21	A+Val/Q+Val
Wang et al. (27)	Comparison	50	30/19	50	11/27	A+Val/Q+Val
Ai et al. (28)	Comparison	48	25/	48	23/	A+Li/Q+Li
Feng et al. (29)	Comparison	54	36/42	54	18/43	A+Li/Q+Li
He et al. (30)	Comparison	81	30/73	81	25/62	A+Li/Q+Li
Wu (31)	Comparison	30	18/29	30	10/22	A+Li/Q+Li
Xu et al. (32)	Comparison	35	13/32	35	8/62	A+Li/Q+Li
Wu (33)	Comparison	28	/23	28	/24	A+Val/Q+Val
Ai et al. (28)	Comparison	32	/21	32	/19	A+Li/Q+Li
Gao et al. (34)	Comparison	43	/23	43	/19	A+Li/Q+Li
Wu et al. (35)	Comparison	28		28		A+Li/Q+Li
Zhang et al. (36)	Comparison	50		50		A+Val/Q+Val
Wang et al. (37)	Comparison	60		60		A+/Q
Wang et al. (28)	Comparison	15		15		A+/Q

AG, Aripiprazole group; QG, Quetiapine group; A, A; Q, Q; Val, valproate salt; Li, lithium carbonate.

### Comparison of remission rate between aripiprazole and quetiapine in the treatment of persons with bipolar depression

**Figure 2:**
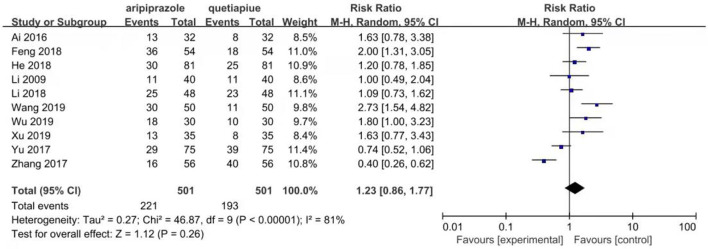
Comparison of remission rate between aripiprazle group and quetiapine group. There were ten studies that reported remission rate.The random effect model was used for analysis of remission rate due to heterogeneity (*X*^2^ = 46.87, *df* = 9, *p* < 0.00001, I^2^ = 81%). The results showed that there was no significant difference in the remission rate between aripiprazole and quetiapine in the treatment of bipolar depression (221/501 vs. 193/501, *Z* = 1.12, *P* = 0.26).

**Figure 3:**
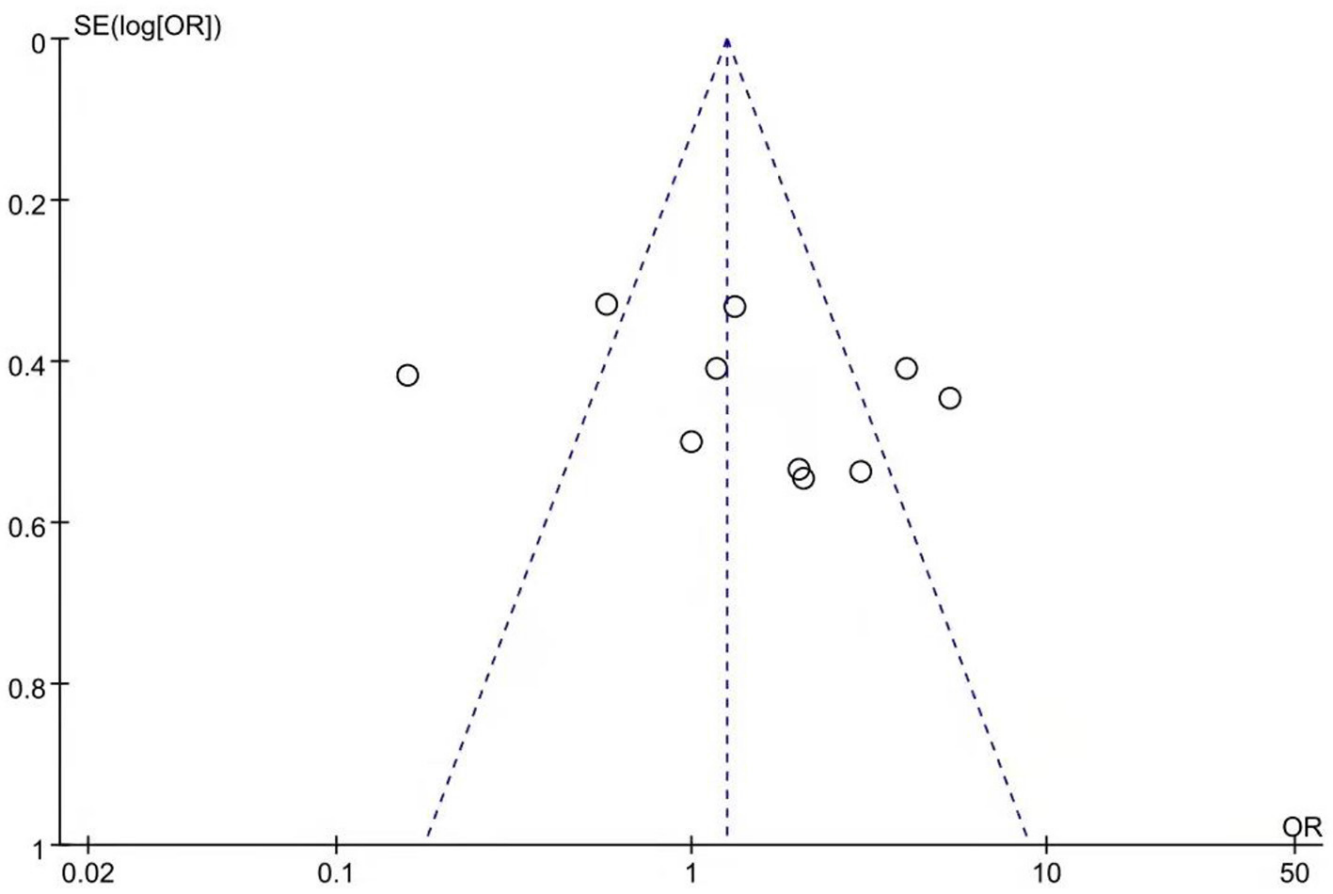
Funnel plot of remission rate comparison between aripiprazole and quetiapine. There was no publication bias in these data.

**Figure 4:**
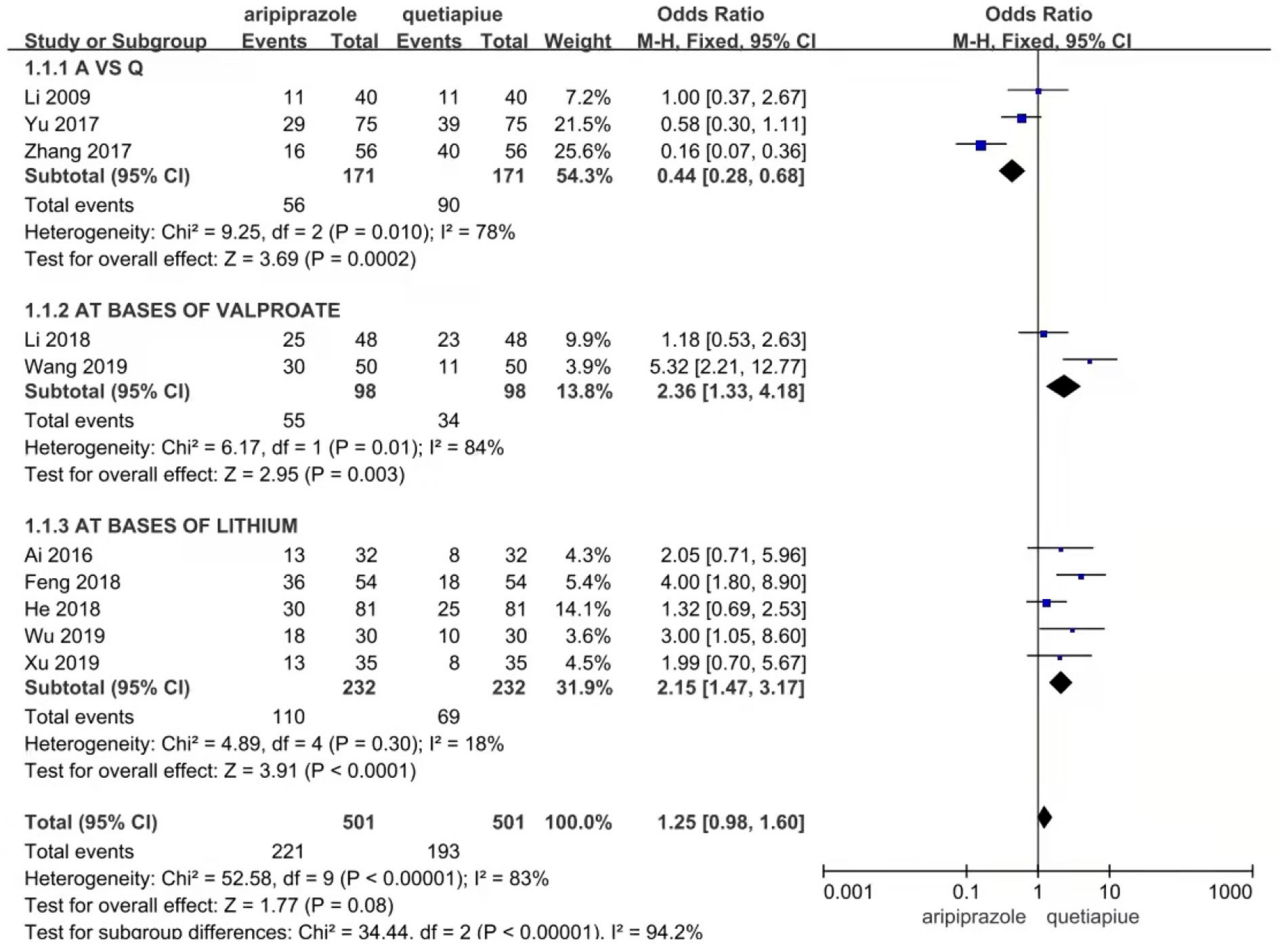
Comparison of remission rate between ariprazole and quetiapine in different subgroup. In case of no combination therapies, the results showed that the remission rate of aripiprazole was significantly lower than quetiapine in the treatment of bipolar depression (56/171 vs. 90/171, Z = 3.69, *P* = 0.0002) by random effect model. In case of combination therapies with valproate sodium, the results showed that the remission rate of aripiprazole was significantly higher than quetiapine in the treatment of bipolar depression (55/98 vs. 34/8, Z = 2.95, *P* = 0.003) by random effect model. In case of combination therapies with lithium carbonate, the results showed that the remission rate of aripiprazole was significantly higher than quetiapine in the treatment of bipolar depression (110/232 vs. 69/232, Z = 3.91, *P* < 0.0001) by fixed effect model.

Ten studies reported remission rates. The random-effects model was used for analysis of remission rate due to heterogeneity (*X*^2^= 52.58, *df* = 9, *p* < 0.01, *I*^2^ = 83%). The results showed that there was no significant difference in the remission rate between patients treated with aripiprazole and quetiapine in the treatment of bipolar depression (221/501 vs. 193/501, Z = 1.12, *P* = 0.26) (see Figure 2). There was no publication bias in the data (see Figure 3). The subgroup comparison was also analyzed. Where monotherapies were used, the results showed that the remission rate of patients treated with aripiprazole was similar to patients treated with quetiapine in the treatment of bipolar depression (56/171 vs. 90/171, Z = 1.73, *P* = 0.08) using the random-effects model. In the case of combination therapies using valproate sodium, the results of the random-effects model showed that the remission rate of patients treated with aripiprazole was significantly higher than patients treated with quetiapine (55/98 vs. 34/8, Z = 1.11, *P* = 0.27). In the case of combination therapies using lithium carbonate, the results of the fixed-effect model showed that the remission rate of patients treated with aripiprazole was significantly higher than patients treated with quetiapine (110/232 vs. 69/232, Z = 3.92, *P* < 0.0001) by (see Figure 4).

### Comparison of effective rate between aripiprazole and quetiapine in the treatment of persons with bipolar depression

**Figure 5:**
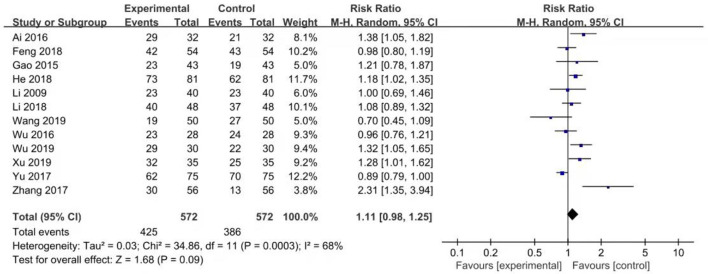
Comparison of effective rate between aripiprazle group and quetiapine group. There were 12 studies that reported effective rate. The random effect model was used for analysis of effective rate due to heterogeneity (*X*^2^ = 34.86, *df* = 11, *p* < 0.0003, *I*^2^ = 68%). The results showed that effective rate of aripripazole was similar to quetiapine in the treatment of bipolar depression (426/572 vs. 386/572, Z = 1.68, *P* = 0.09).

**Figure 6:**
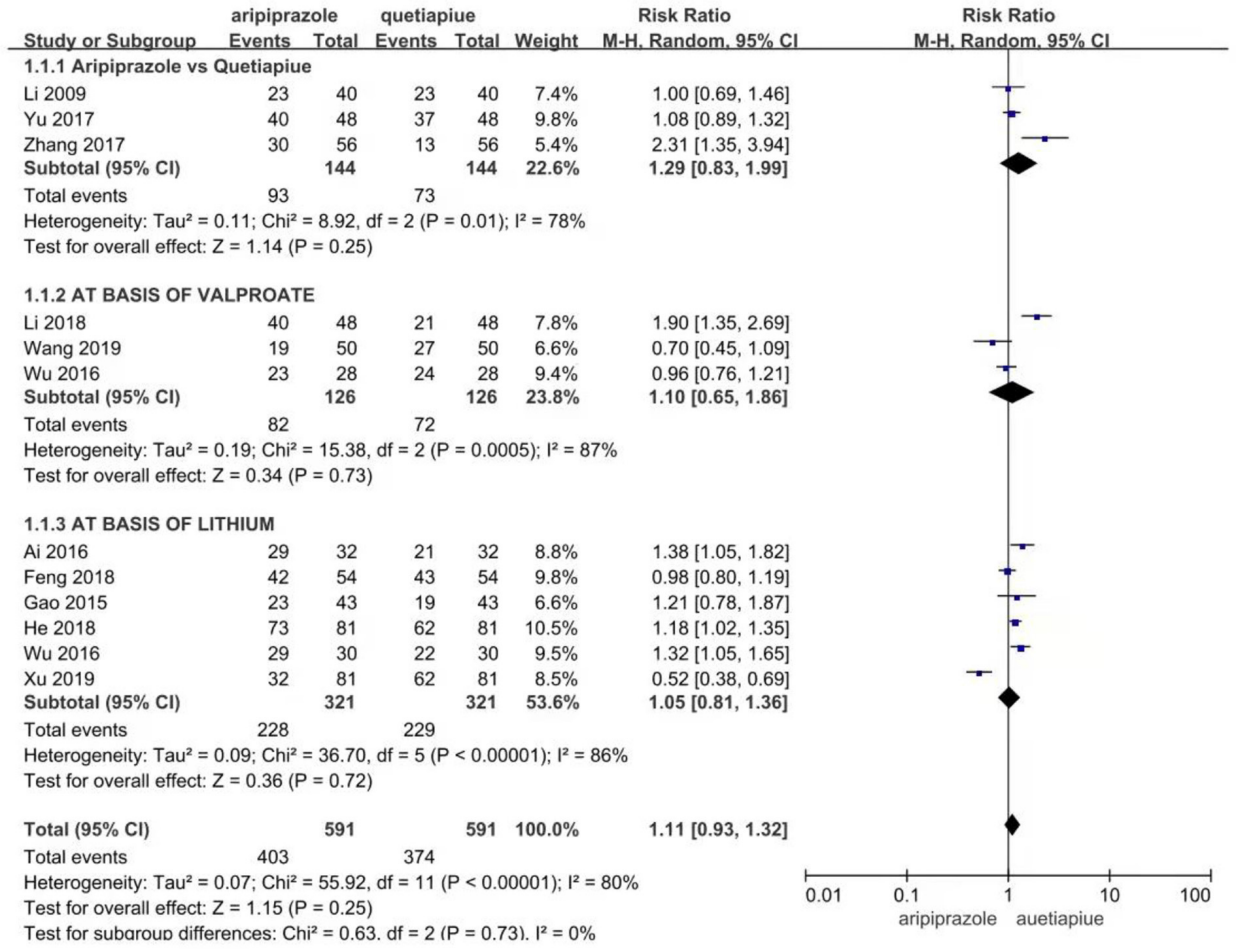
Comparison of effective rate between aripiprazle and quetiapine in different subgroup. In case of no combination therapies, the results also still showed that the effective rate of aripiprazole in the treatment of bipolar depression was similar to quetiapine (93/144 vs. 73/144, Z = 1.14, *P* = 0.251) by random effect model. However, in the combination therapies with mood stabilizer of valproate salt, the effective rate of aripiprazole war smilar to quetiapine in the treatment of bipolar depression (82/126 vs. &2/126, Z = 0.34, *P* = 0.72) by random effect model. In the combination therapies with mood stabilizer of lithium carbonate, the effective rate of aripiprazole war similar to quetiapine in the treatment of bipolar depression (228/321 vs. 229/321, Z = 0.36, *P* = 0.72) by random effect model.

**Figure 7:**
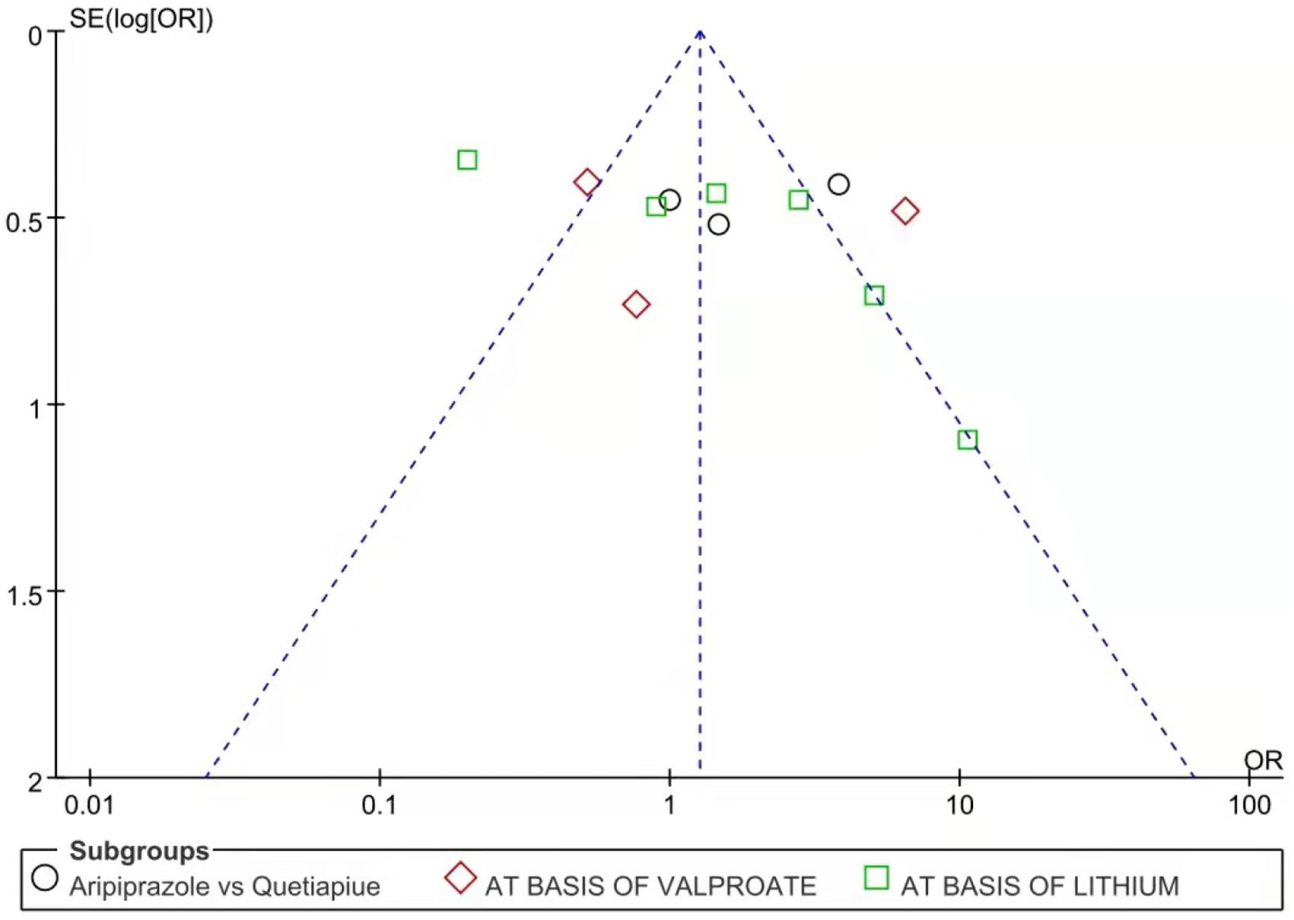
Funnel plot of effective rate between aripiprazole and quetiapine in different case. There was no publication bias in these data.

Twelve studies reported effective rates. The random-effects model was used for the analysis of the effective rate due to heterogeneity (*X*^2^ = 31.81, *df* = 11, *p* < 0.01, *I*^2^ = 65%). The results showed that the effective rate of aripiprazole was similar to quetiapine in the treatment of bipolar depression (426/572 vs. 386/572, Z = 1.68, *P* = 0.09) (see Figure 5). In the case of monotherapies, the results showed that the effective rate of aripiprazole in the treatment of bipolar depression was similar to quetiapine (93/144 vs. 73/144, Z = 1.14, *P* = 0.25) using the random-effects model. However, in combination therapies using mood stabilizers such as valproate sodium, the effective rate of quetiapine was similar to aripiprazole (82/126 vs. 72/126, Z = 0.34, *P* = 0.73) by the random-effects model. In the combination therapies using lithium carbonate, the effective rate of quetiapine was similar to aripiprazole (228/321 vs. 229/321, Z = 0.36, *P* = 0.72) by the random-effects model (see Figure 6). There was no publication bias in these data (see Figure 7).

### Comparison of HAMD scale between aripiprazole and quetiapine in different therapeutic stages and overall treatment

**Figure 8:**
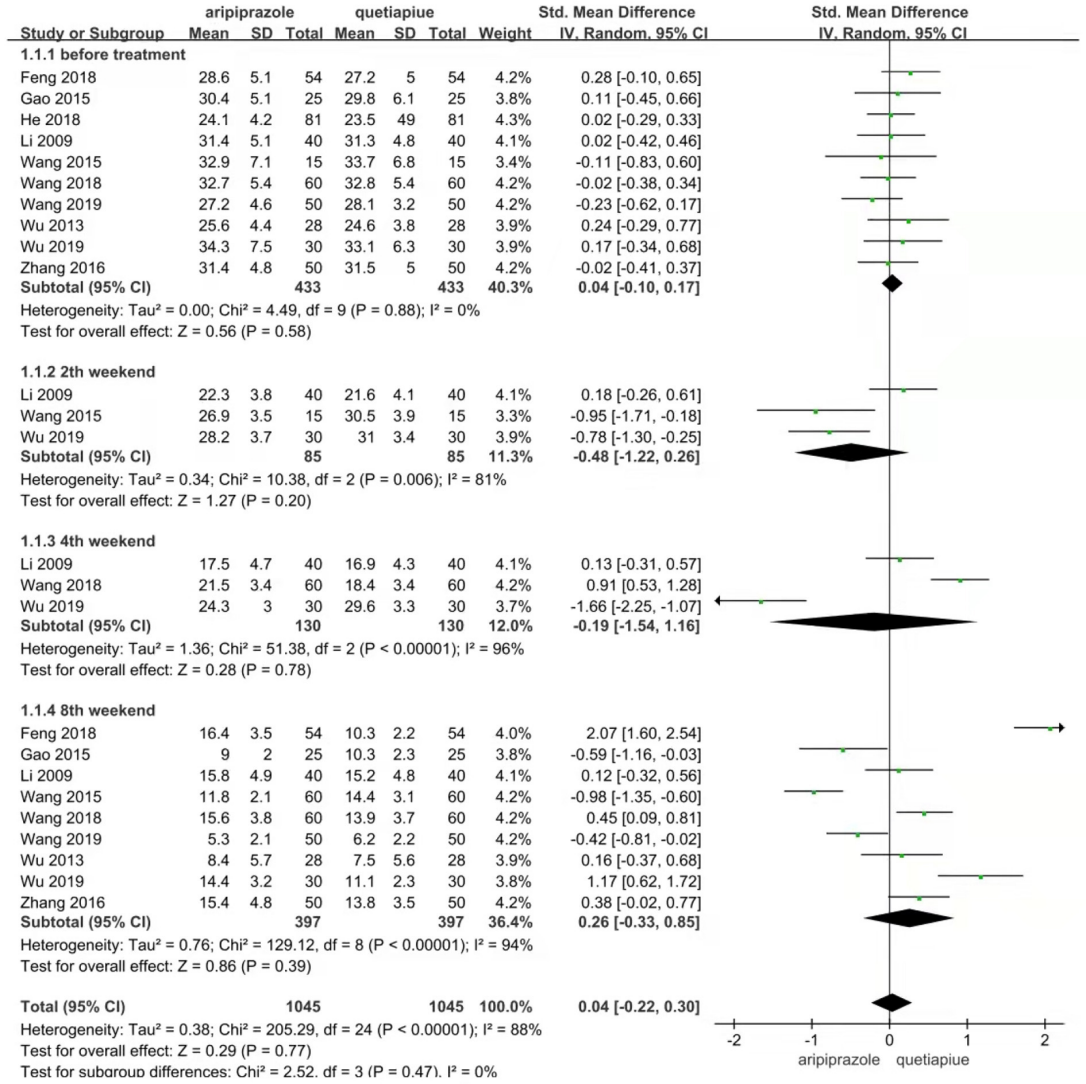
Comparison of HAMD scale between aripripazole and quetiapine in different therapeutic stage. Comparison of HAMD scale were analyzed between aripripazole and quetiapine by random effect model due to their heterogeneity (*X*^2^ = 205.29, df = 24, *P* < 0.00001, *I*^2^ = 88%). Overall there were no difference in HAMD scale between aripripazole and quetiapine before treatment (Z = 0.29, *P* = 0.77). The HAMD scale of aripiprazole group was silmilar to quetiapine at 2nd weekend (Z = 1.273, *P* = 0.20). The HAMD scale of aripiprazole was similar to quetiapine at 4th weekend (Z = 0.28, *P* = 0.78). The HAMD scale of aripiprazole was significantly higher than that of quetiapine at 8th weekend (Z = 0.86, *P* = 0.39).

The HAMD Scale Was Compared Between the Aripiprazole and Quetiapine Groups Using the Random-Effects Model due to Their Heterogeneity (X^2^ = 205.29, df = 24, *P* < 0.00001, I^2^ = 88%). Overall, There Was no Difference in the HAMD Scale Between the Aripiprazole and Quetiapine Groups Before Treatment (Z = 0.29, *P* = 0.77). The HAMD Scale of the Aripiprazole Group Was Similar to the Quetiapine Group on the Second Weekend (*Z* = 1.27, *P* = 0.20) and Fourth Weekend (Z = 0.28, *P* = 0.78). However, on the Eighth Weekend, the HAMD Scale of the Aripiprazole Group Was Significantly Higher Than That of the Quetiapine Group (Z = 0.86, *P* = 0.39). At end of the Study, It Was Noticed That Quetiapine Played a Role Similar to Aripiprazole (see Figure 8).

### Comparison of the drop-out rate between the aripiprazole and quetiapine groups

**Figure 9:**
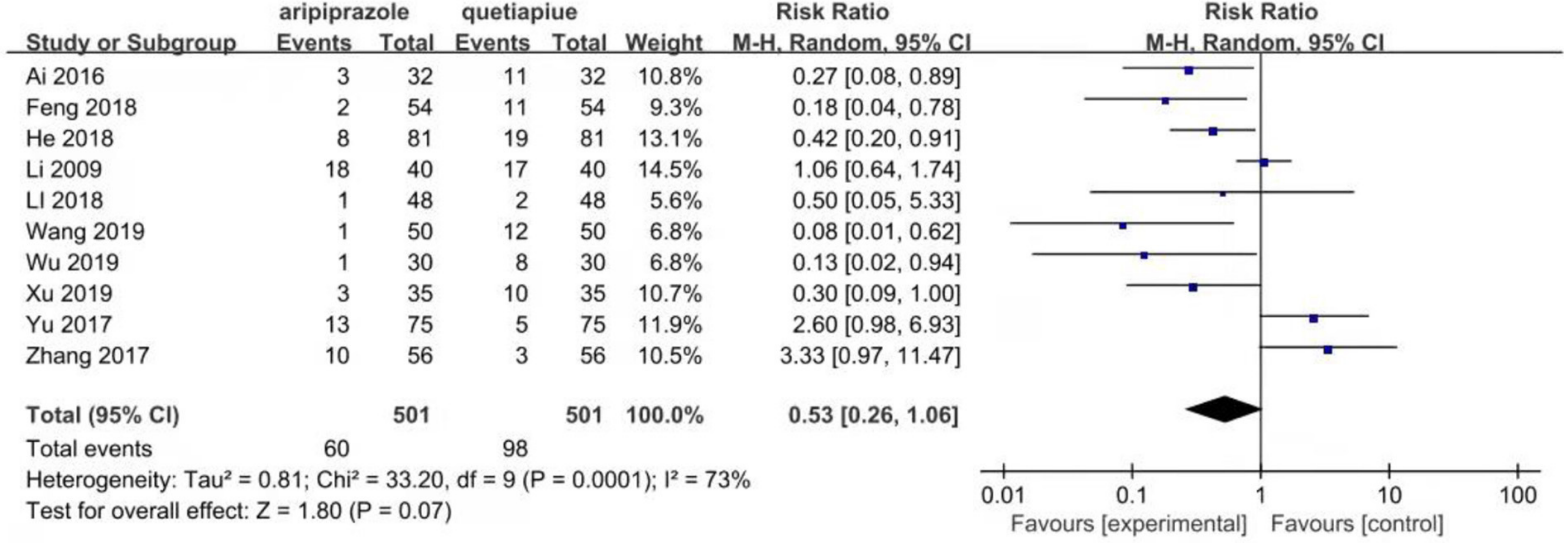
Comparison of the drop out rate between aripripazole and quetiapine group. The drop out rate is an indicator of drug side effects or acceptance to drug, which was analyzed by random effect model due to their heterogeneity (*X*^2^ = 33.20, *df* = 9, *P* = 0.0001, *I*^2^ = 73%). The results show that drop out rate of aripripazole was similar to quetiapine (Z = 1.80, *P* = 0.07).

The drop-out rate is an indicator of drug side effects or acceptance of the drug, which was analyzed by the random-effects model due to their heterogeneity (*X*^2^ = 33.20, *df* = 9, *P* = 0.0001, *I*^2^ = 73%). The results show that the drop-out rate of aripiprazole was similar to quetiapine (*Z* = 1.80, *P* = 0.07) (see Figure 9).

## Discussion

Aripiprazole, as a partial dopamine receptor agonist, has been approved by FDA for the treatment of schizophrenia and mania. However, there is no reliable evidence for the use of aripiprazole in the treatment of bipolar depression (40). Overall, available randomized control trial (RCT) studies seem to support the hypothesis that the augmentation with second-generation antipsychotics (SGAs), in particular aripiprazole and quetiapine, is a valid therapeutic option for treatment-resistant depression (18). Extending the same hypothesis, aripiprazole was also introduced into the treatment of bipolar depression. Clinically, aripiprazole monotherapy at the doses studied may provide some improvements in core symptoms of depression in persons with bipolar I disorder who are more severely depressed (17). In the early stages, the meta-analysis found evidence suggesting that aripiprazole is effective in both depressive and manic patients, but has relevant side effects (41). Another meta-analysis also found that aripiprazole combination therapy with lithium carbonate was more effective than just lithium carbonate in patients with bipolar depression (42). In recent years, more studies in China are using aripiprazole in the treatment of bipolar depression and comparing it with quetiapine (23–39).

Atypical antipsychotic quetiapine, which is used for schizophrenia and organic psychosis, depression with psychotic symptoms can not only treat treatment-resistant depression but also treat bipolar depression (8, 12, 14, 15). Therefore, quetiapine has always been the main force in the treatment of bipolar depression. In recent years, Lurasidone and Cariprazine have been suggested for treating bipolar depression in therapy guides and clinical practice (8, 13, 17). But the use of aripiprazole still raises questions because there is no reliable evidence of its treatment of bipolar depression. Although aripiprazole has proven efficacy for acute mania and the prevention of mania, the evidence available thus far does not support the efficacy of aripiprazole for the treatment of acute bipolar depression and prevention of depressive relapse (40). So further evaluations comparing aripiprazole and quetiapine in the therapy of bipolar depression may be required to provide evidence for aripiprazole in treatment for bipolar depression.

This study is a meta-analysis of aripiprazole and quetiapine in the treatment of bipolar depression undertaken by Chinese psychiatrists (23–39). Based on these studies, we suggest that there is no significant difference between aripiprazole and quetiapine in remission rate, effective rate, and depression symptom score, but the drop-out rate of aripiprazole is significantly lower than quetiapine, although with no statistical significance. This conclusion seems to indicate that aripiprazole, like quetiapine, can treat bipolar depression, and the drop-out rate is relatively low. Although minor differences in different cases exist, aripiprazole has some advantages, especially in a higher remission rate in the case of combination therapies using lithium carbonate.

This study did reveal some differences between aripiprazole and quetiapine in the treatment of bipolar depression. In the case of combination therapies with lithium carbonate, the results using the fixed-effects model showed that the remission rate of aripiprazole was significantly higher than quetiapine in the treatment of bipolar depression (110/232 vs. 69/232, Z = 3.92, *P* < 0.0001). However, this may not affect the use of aripiprazole in the treatment of bipolar depression, but it calls for more caution while including aripiprazole into the treatment guidelines of bipolar depression.

The evaluation of side effects is an important part of the study of clinical efficacy. This study evaluated the drop-out rate related to side effects, compliance, insight, and other factors. Fortunately, aripiprazole has good advantages, which have been confirmed in other similar research (43), but some studies have put forward opposite views that aripiprazole showed higher discontinuation rates vs. placebo due to the appearance of any adverse event (44), which we should pay attention to. In the study of side effects, switching to mania is also worthy of attention (45). Unfortunately, this study did not pay attention to this phenomenon.

The psychopharmacological mechanisms of aripiprazole and quetiapine are different. Aripiprazole is a derivative of the dopamine autoreceptor agonist and is a third-generation antipsychotic with a dopamine receptor-binding profile distinct from other second-generation antipsychotics. It acts as a partial agonist at dopamine D2 and 5-hydroxytryptamine (5-HT)1A receptors, stabilizing the dopamine receptor and leading to improvement in symptoms, which may be the biological factor associated with better therapeutic efficacy (11, 16, 18). Quetiapine is an atypical antipsychotic drug, which involves blocking multiple receptors including the D2 receptor. The antidepressant activity of quetiapine is mediated, at least in part, by the active metabolite norquetiapine, which selectively inhibits noradrenaline reuptake. It is a partial 5-HT1A receptor agonist and acts as an antagonist at presynaptic α2, 5-HT2A, and 5-HT7 receptors (46). It may be related to the antagonism of 5-HT2A receptors in cortical regions, and partial agonism of 5-HT1A in the pre-frontal cortex in association with increased extracellular dopamine release in the region. Conversely, it may also be related to reduced synaptic reuptake of noradrenaline resulting from inhibition of the noradrenaline reuptake transporter by the quetiapine metabolite norquetiapine (47). However, this does not lead to their overall differences in this study. It also shows that the mechanisms of aripiprazole and quetiapine in the treatment of bipolar depression may be different.

## Conclusion

As an atypical antipsychotic, aripiprazole may be similar to quetiapine for treating bipolar depression with some advantages of less drop-out rate and a higher effective rate. But, given the limited literature on the subject, and that too of poor quality, the results of this study need to be read with caution.

## Limitation

The shortcomings of this study are the following: First, there are many studies in which the quality of the collected literature is not very good, and there is almost no standard random control trial (RCT) studies. Second, various side effects were not evaluated and analyzed. Third, the results of this study provide limited evidence for aripiprazole to be written into the guidelines for the treatment of bipolar depression. Therefore, we need to design more rigorous RCT studies to carefully evaluate the similarities and differences between aripiprazole and quetiapine in the treatment of bipolar depression, especially in a psychiatric clinic in China.

## Data availability statement

The original contributions presented in the study are included in the article/supplementary material, further inquiries can be directed to the corresponding author.

## Author contributions

HT and WJ participated in the collection of data and the writing of the article. WJ and FS assessed the quality of the researched papers. HT undertook most of the statistical analysis. JW participated in the design, statistical processing, and final revision of the article. All authors reviewed all researched papers.

## Funding

This study was supported by PSP-2022-010 (Peak Subject of Psychiatry, Zhejiang Province Tongde Hospital) in Design, Literature Collection, and Statistic Analysis.

## Conflict of interest

The authors declare that the research was conducted in the absence of any commercial or financial relationships that could be construed as a potential conflict of interest.

## Publisher's note

All claims expressed in this article are solely those of the authors and do not necessarily represent those of their affiliated organizations, or those of the publisher, the editors and the reviewers. Any product that may be evaluated in this article, or claim that may be made by its manufacturer, is not guaranteed or endorsed by the publisher.

## Abbreviations

MEDLINE: The National Library of Medicine
EMBASE: Excerpta Medical Database
CBM: Chinese Biomedical Database
CNKI: China National Knowledge Infrastructure
CSSCI: Chinese Social Sciences Citation Index (VIP)
HAMD: Hamilton Depression Scale
TRD: treatment-resistant depression
BD: Bipolar disorder.
